# Bioinspired Artificial Intelligence Applications 2023

**DOI:** 10.3390/biomimetics9020080

**Published:** 2024-01-28

**Authors:** Haoran Wei, Fei Tao, Zhenghua Huang, Yanhua Long

**Affiliations:** 1 Department of ECE, University of Texas at Dallas, Richardson, TX 75081, USA; haoran.w@samsung.com; 2 Amazon AWS, Seattle, WA 98109, USA; fei.tao@hotmail.com; 3 School of Electrical and Information Engineering, Wuhan Institute of Technology, Wuhan 430205, China; zhhuang@wit.edu.cn; 4 The College of Information, Mechanical and Electrical Engineering, Shanghai Normal University, Shanghai 200234, China

With rapid development of Artificial Intelligence (AI), researchers have found many bioinspired AI applications, such as bioinspired images and speech processing, which can increase accuracy [1,2,3]; bioinspired AI models deployed on edge devices can reduce cost and energy usage [4]; bioinspired AI noise reduction technologies could enhance the bio-signal quality [5,6,7,8]; smart watch-generated bio-signals can be utilized by AI to monitor users’ health condition [9,10], and so on.

On the other hand, improper AI utilization also creates challenges for human beings [11,12,13], such as synthesised images by AI can be used to generate fake news, AI synthesised speech can simulate a target speaker and cause security issues for personal devices, AI generated speech and text can be used for phone scams, and so on.

To explore the potential of AI applications and also deal with these challenges for improper AI utilization, state-of-the-art AI-based technologies in image processing, video processing, speech and audio processing, natural language processing, multi-modality processing, internet-of-things, edge computing, autonomous deriving, and smart healthcare, can adding intelligence to human-centered AI applications and solved the challenges associated with improper AI utilization.

The aim of this Special Issue is to present a multidisciplinary state-of-the-art reference regarding theoretical and real-world challenges, and innovative solutions by inviting high-quality research papers for bioinspired AI applications.

This Special Issue collects contributions from different laboratories and research institute working on Bioinspired Artificial Intelligence Applications. Paper [14] utilized deep reinforcement learning to deal with the robot task sequencing problem and trajectory planning problem. This method model the optimization process as a Markov decision process and propose a deep reinforcement learning (DRL)-based method to facilitate problem solving.The real-world experimental results demonstrate that the DRL method can achieve a 30.54% energy savings compared to the traditional evolution algorithm, and the computational time required by the proposed DRL method is much shorter than those of the evolutionary algorithms. Paper [15] proposed YOLOv5-MS, a real-time multi-surveillance pedestrian target detection model, to address challenges about slow detection speeds and increased costs. The proposed model optimize the multi-threaded acquisition of video streams within YOLOv5 to ensure image stability and real-time performance. For leveraging reparameterization, the prospoed model replace the original BackBone convolution with RepvggBlock, streamlining the model by reducing convolutional layer channels, thereby enhancing the inference speed. Additionally, the incorporation of a bioinspired “squeeze and excitation” module in the convolutional neural network significantly enhances the detection accuracy. Furthermore, the integration of the K-means algorithm and bioinspired Retinex image augmentation during training effectively enhances the model’s detection efficacy. Finally, loss computation adopts the Focal-EIOU approach. Paper [16] proposed an intelligent breast mass classification approach using archimedes optimization algorithm with deep learning (BMCA-AOADL) ton digital mammograms. The major aim of the BMCA-AOADL technique is to exploit the deep learning model with a bio-inspired algorithm for breast mass classification. In the BMCA-AOADL approach, median filtering (MF)-based noise removal and U-Net segmentation take place as a pre-processing step. For feature extraction, the BMCA-AOADL technique utilizes the SqueezeNet model with AOA as a hyperparameter tuning approach. To detect and classify the breast mass, the BMCA-AOADL technique applies a deep belief network (DBN) approach. The simulation value of the BMCA-AOADL system has been studied on the MIAS dataset from the Kaggle repository. The experimental values showcase the significant outcomes of the BMCA-AOADL technique compared to other deep learning algorithms with a maximum accuracy of 96.48%. Paper [17] proposed a bio-inspired object detection algorithm for remote sensing images. Due to the small size of the target, complex background information, and multi-scale remote sensing images, the generalized YOLOv5 detection framework is unable to obtain good detection results. To deal with these issues, this paper proposed YOLO-DRS, a bioinspired object detection algorithm for remote sensing images incorporating a multi-scale efficient lightweight attention mechanism. Compared to the state-of-the-art algorithms, namely YOLOv8s and YOLOv7-tiny, the proposed approach demonstrates significant improvements in the mAP@0.5 metrics, with enhancements ranging from 1.8% to 7.3%. It is fully proved that proposed YOLO-DRS apporoach can reduce the missed and false detection problems of remote sensing target detection. Paper [18] utilized bio-inspired artificial intelligence with natural language processing approach to detect the presence of deceptive or fake content on social media. The proposed approach applies data preprocessing to transform the input dataset into a meaningful format, then uses a multi-head self-attention bi-directional long short-term memory (MHS-BiLSTM) model for deceptive content detection. After that, the African Vulture Optimization Algorithm (AVOA) is applied for the selection of optimum hyperparameters of the MHS-BiLSTM model.

Taking advantage of its open access publication, this collection of papers, influenced by biomimetic approaches, will bring about new avenues for new research and innovative solutions in biomedicine and technology. We believe that this special issue may fulfill an important function in promoting biomimetic artificial intelligence for an increasing range of applications.
